# Targeting the TGFβ pathway with galunisertib, a TGFβRI small molecule inhibitor, promotes anti-tumor immunity leading to durable, complete responses, as monotherapy and in combination with checkpoint blockade

**DOI:** 10.1186/s40425-018-0356-4

**Published:** 2018-06-04

**Authors:** Rikke B. Holmgaard, David A. Schaer, Yanxia Li, Stephen P. Castaneda, Mary Y. Murphy, Xiaohong Xu, Ivan Inigo, Julie Dobkin, Jason R. Manro, Philip W. Iversen, David Surguladze, Gerald E. Hall, Ruslan D. Novosiadly, Karim A. Benhadji, Gregory D. Plowman, Michael Kalos, Kyla E. Driscoll

**Affiliations:** 1 Lilly Research Laboratories, Eli Lilly and Company, Indianapolis, IN 46285 USA; 2 Eli Lilly and Company, 450 East 29th Street, New York, USA; 3 Janssen Pharmaceutical Companies of Johnson and Johnson, Spring House, PA USA

**Keywords:** TGF-β receptor I, Antitumor efficacy, Checkpoint inhibitors, Galunisertib

## Abstract

**Background:**

TGFβ signaling plays a pleotropic role in tumor biology, promoting tumor proliferation, invasion and metastasis, and escape from immune surveillance. Inhibiting TGFβ’s immune suppressive effects has become of particular interest as a way to increase the benefit of cancer immunotherapy. Here we utilized preclinical models to explore the impact of the clinical stage TGFβ pathway inhibitor, galunisertib, on anti-tumor immunity at clinically relevant doses.

**Results:**

In vitro treatment with galunisertib reversed TGFβ and regulatory T cell mediated suppression of human T cell proliferation. In vivo treatment of mice with established 4T1-LP tumors resulted in strong dose-dependent anti-tumor activity with close to 100% inhibition of tumor growth and complete regressions upon cessation of treatment in 50% of animals. This effect was CD8+ T cell dependent, and led to increased T cell numbers in treated tumors. Mice with durable regressions rejected tumor rechallenge, demonstrating the establishment of immunological memory. Consequently, mice that rejected immunogenic 4T1-LP tumors were able to resist rechallenge with poorly immunogenic 4 T1 parental cells, suggesting the development of a secondary immune response via antigen spreading as a consequence of effective tumor targeting. Combination of galunisertib with PD-L1 blockade resulted in improved tumor growth inhibition and complete regressions in colon carcinoma models, demonstrating the potential synergy when cotargeting TGFβ and PD-1/PD-L1 pathways. Combination therapy was associated with enhanced anti-tumor immune related gene expression profile that was accelerated compared to anti-PD-L1 monotherapy.

**Conclusions:**

Together these data highlight the ability of galunisertib to modulate T cell immunity and the therapeutic potential of combining galunisertib with current PD-1/L1 immunotherapy.

## Background

Transforming growth factor-beta (TGFβ) has been identified as a therapeutic target in cancer because of its significant and varied roles to promote tumor growth, survival, and metastasis. There are several pharmacological approaches to block TGFβ signaling, including neutralizing antibodies, vaccines, antisense oligonucleotides and small molecular inhibitors (SMI) [1, 2]. The goal of these therapies is to block the tumor-promoting effects of TGFβ, while maintaining its tumor suppressive properties. Emerging data and thought suggest that the efficacy of TGFβ antagonist therapy in cancer might not only derive from direct intrinsic effects on tumor cells, but also involves tumor extrinsic mechanisms acting in the tumor micro-environment.

TGFβ plays pleiotropic roles to initiate and progress cancer including both tumor cell intrinsic and extrinsic activities. Tumor cell intrinsic activities of the TGFβ pathway include autocrine TGFβ driven tumor cell proliferation and differentiation, epithelial to mesenchymal transition (EMT), invasion and migration, prometastatic cytokine production, and autocrine mitogen production [3, 4]. Tumor cell extrinsic activities include promoting of increased tumor vascularization, modulation of the stromal extracellular matrix, induction of and feedback modulation of the hypoxic state and inhibition of immune surveillance and antitumor immunity [4, 5].

Systemic TGFβ ligand levels are often elevated in cancer patients compared to healthy individuals, and increased ligand levels have been further associated with aggressive disease and poor prognosis [6, 7]. Elevated TGFβ ligand levels are observed in patients whose tumor cells are both sensitive (i.e. receptor positive, TGFβ ligand dependent) or insensitive (i.e receptor negative, TGFβ ligand independent) to TGFβ signaling. Furthermore, aberrant TGFβ signaling has been implicated in several human diseases, including malignancies such as glioblastoma and breast cancer [8–10].

TGFb additionally plays a non-redundant, crucial role in regulating immunity. TGFβ is produced by a number of immune cells and plays an essential role in the regulation of immune responses and immune tolerance [4, 11]. Genetic deletion and antibody neutralization studies have demonstrated that TGFβ inhibition enhances T cell [12] and NK cell differentiation and function [13], suggesting that pharmacologic inhibition of TGFβ signaling might decrease the suppression of host immune surveillance. Furthermore, deletion of TGFβ signaling in myeloid cells has been shown to enhance their anti-tumorigenic properties [14]. The immunological consequences of TGFβ antagonism are particularly relevant in the context of anti-tumor immunotherapy, and blockade of the TGFβ pathway has become an attractive approach to inhibit the multitude effects the TGFβ pathway has on cancer progression and anti-tumor immunity. That TGFβ may be involved in the maintenance of self-tolerance and pathogenesis of systemic inflammatory diseases is indicated in studies which show the development of multi-organ inflammation in *Tgfb1*^−/−^ mice [15, 16]. The inflammation in *Tgfb1*^−/−^ mice is dependent on T cells, which undergo massive activation [17]. Generation of mice lacking TGFβRII specifically on T-cells further demonstrates the importance of TGFβ in regulating T-cell responses in vivo, as mice develop multi-organ inflammation similar to that seen in *TGFβ1−/−* mice [12, 18].

In addition to the direct effects on effector T cell responses, TGFβ can promote immunosuppression via direct induction and modulation of regulatory T cells (Tregs) [19]. TGFβ directly promotes expression of Foxp3 in CD4^+^ T-cells, converting them to a regulatory phenotype [20]. In addition to induction and maintenance of Foxp3 expression, TGFβ has also been shown to be important in the functional ability of Tregs to suppress immune responses [21, 22], and it has been demonstrated that *Tgfb1−/−* mice fail to maintain peripheral Treg cells [21]. TGFβ1-producing myeloid-derived suppressor cells (MDSCs) have also been reported at high levels in the tumor microenvironment [23, 24].

Clinical studies have provided proof of concept data supporting the role of TGFβ in cancer and the utility of targeting the TGFβ pathway [1]. Galunisertib (LY2157299 monohydrate) is an oral small molecule inhibitor (SMI) of the TGFβ receptor I (TGFβRI) kinase that specifically downregulates the phosphorylation of SMAD2, abrogating activation of the canonical pathway [1] (Yingling et al., [25]). By targeting TGFβRI, signaling via all three TGFβ ligands is blocked [1]. Galunisertib demonstrates the ability to inhibit TGFβ-dependent tumor cell intrinsic and extrinsic functions in vitro and in vivo, and to inhibit tumor-cell growth in established tumor mouse models (Yingling et al., [25]). Galunisertib is currently under clinical development in combination with checkpoint inhibitors (including nivolumab and durvalumab) in patients with NSCLC, HCC, or pancreatic cancer (NCT02423343; NCT02734160).

In the current study, we set out to characterize in detail the impact of galunisertib-mediated TGFβR1 blockade on anti-tumor immunity. Using both in vitro and in vivo model systems, we show that galunisertib enhances the development of anti-tumor T cell immunity through modulating both effector and regulatory T cell function. Using an immunogenic 4 T1-LP breast tumor model, we show that galunisertib mediates robust anti-tumor T cell immunity and promotes the establishment of T cell memory and antigen spreading. Using in vitro assays and primary human Treg cells we show that Galunisertib treatment blocks the suppressive activity of human Tregs, further highlighting its important role in T cell immunity. The TGFβ pathway was recently described as a potential mechanism of resistance for anti-PD-1/L1 checkpoint blockade [26, 27]. To this end, we show that galunisertib treatment at a clinically relevant dose enhances the anti-tumor activity of anti-PD-L1 resulting in robust tumor regressions associated with enhanced T-cell activation signatures, further supporting the clinical development of targeting TGFβRI in combination with checkpoint blockade. Clinical trials evaluating galunisertib in combination with anti-PD-1 immunotherapy are currently being conducted (https://clinicaltrials.gov; NCT02734160 and NCT02423343) and thus, gives this research a highly translational impact.

## Methods

### Human CD8 T cell suppression assays with TGFβ

CD8^+^ T cells were purified from healthy donor blood (New York Blood Center, NY, NY) with RosetteSep Human CD8^+^ T cells enrichment kit (Stemcell Technologies) and labeled with 1 mM CFSE (Invitrogen) in pre-warmed PBS+5%FCS for 10 min at 37 °C. Cells were then plated onto 96-well plates (5 × 10^4^/well) in complete RPMI media (Gibco) and stimulated with human T cell activation/expansion beads (Miltenyi Biotech). Cells were cultured with or without TGFβ1 at 10 ng/ml. Galunisertib was added at indicated concentration (0.1μM to 10 μM) with DMSO as vehicle control. Percent CD8 T cell proliferation was measured by assessing CFSE dilution by FACS (BD LSRFortessa) after 5 days of culture. Recovery of T cell proliferation was calculated according to the formula: % of Max proliferation = % CFSE low of sample/(average of CFSE low for control with no TGFβ). One-way ANOVA followed by Dunnett’s test was performed to assess statistical significance.

### Human Treg suppression assay

CD4^+^ cells purified from heathy donor blood (New York Blood Center, NYC) using the Rosetta CD4^+^ T cell enrichment kit (Stem Cell Technologies). CD25^+^ and CD25^−^ T cells were then isolated using human CD25^+^ T cell microbeads (Miltenyi). Naïve CD25^−^ T cells were labeled with 1 mM CFSE (Invitrogen) as described above. CD25^−^ naïve T cells and CD25^+^ Tregs were re-suspended in complete RPMI media (Gibco) and plated onto 96-well plates at indicated ratios of Treg cells to naïve T cells with 5 × 10^4^ cells/well in total; except for Tregs alone and untreated naïve T cells which were plated at 2.5 × 10^4^ cells/well. Cells were then stimulated with CD3/CD28/CD2 antibody coated beads (Miltenyi) at a bead to cell ratio of 1:1with unstimulated CD25^−^ naïve T cells as a control. Galunisertib (0.1μM to 10 μM) was added with DMSO as vehicle control. Proliferation was measured by CFSE dilution as above after 5–7 days of culture. Rescue of proliferation was calculated according to the formula: Percent recovery of proliferation = (%CFSE low T naive in treated Treg co-culture - %CFSE low T naive in untreated Treg co-culture)/(% CFSE low untreated T naive monoculture stimulated with beads - %CFSE low T naive in untreated Treg co-culture) × 100%. One-way ANOVA followed by Dunnett’s test was performed to assess statistical significance.

### Murine cell lines

CT26.WT (CT26) colon and 4 T1 and EMT6 breast tumor lines, were purchased from American Type Culture Collection (ATCC; Manassas, VA). MC38 colon tumor cell line was purchased from the NCI tumor repository (Frederick, MD). The 4T1 luciferase positive (4T1-LP) cell line was developed at Lilly NYC from the 4T1 parental cell line stably transduced with firefly luciferase (luciferase plasmid pLXSN-luc, G418). The EMT-6-LM2 was generated following serial passage of metastatic parental EMT6 cells [13].

### Mice

Female Balb/c (WT and Rag^−/−^) and C57BL/6 mice (6 to 8 weeks of age) were purchased from Harlan Laboratories/Envigo. All experimental procedures were done in accordance with the guidelines of the NIH “Guide for Care and Use of Animal” and approved protocols reviewed by Institutional Animal Care and Use Committee.

### In vivo studies: Tumor challenge and treatment experiments

4T1 and 4T1-LP tumors were generated by injection of 1 × 10^6^ cells orthotopically in the mammary fat pad of Balb/C mice. Galunisertib was dosed P.O. at 37.5 mg/kg, 75 mg/kg or 150 mg/kg twice daily (BID) for 28 days, with HEC (1% hydroxyethyl cellulose (HEC) in 25 mM phosphate buffer, pH = 2) as control vehicle. For combination therapy studies, 1 × 10^6^ CT26 or 5 × 10^5^ MC38 cells were injected subcutaneously into the flank of Balb/c or C57BL/6 mice, respectively. Galunisertib was dosed at 75 mg/kg BID for 21 days and anti-PD-L1 antibody (clone 178G7; Lilly NYC) or Rat IgG antibody was given 3 times intraperitoneal at 500μg/dose every 7 days (q7dx3). For depletion of CD8 T cells, mice were injected i.p. with 200 μg of CD8a antibody (clone 53–6.7; eBioscience) on day 1, 2 and 3 after tumor challenge, followed by injection of 200 μg weekly throughout the experiment. For all studies, mice were randomized by body weight or tumor volume into groups of 8–15 mice prior to treatment. For MOA experiments, separate subgroups of 3–5 animals/MOA timepoint were pre-assigned at study initiation and not included in survival evaluation.

Tumor volume was calculated using a formula: Tumor Volume (mm^3^) = π/6 * Length * Width^2^. Animals were sacrificed due to progressive disease if tumor burden was greater than 2500 mm^3^ and if growth would surpass 2500 mm^3^ before the next scheduled measurement. For rechallenge experiments mice with complete regressions (tumor volume < 14 mm^3^) were rechallenged as indicated and followed for ~ 30 days.

Tumor volumes compared to control (%T/C) were calculated as %T/C = 100 x ∆T/∆C, whereby ∆T = mean tumor volume of treated group, and ∆C = mean tumor volume of the control (vehicle) on indicated day minus the mean tumor volume on the baseline. Statistical analysis was performed by two-way repeated measures analysis using the log transformation of tumor volume. Predefined pairwise comparisons were conducted as indicated.

### Isolation of tumor-infiltrating cells and lymphoid tissue cells

Tumors and spleens were harvested from individual mice at specific MOA time points after tumor cell inoculation. Single cell suspensions were made by homogenizing each tissue separately through 40 μm nylon mesh strainers into complete media (RPMI+ 10% FBS). After RBC lysis (ACK lysis buffer; Gibco) when required, all samples were washed and re-suspended in FACS buffer (PBS + 4% BSA) for fresh FACS analysis or snap frozen for gene expression analysis.

### FACS analysis

Single cell suspensions prepared from mouse tumors and spleens were pre-incubated with 1 μl/ml anti-CD16/32 monoclonal antibody (Fc block; Tonbo) for 30 min at 4 °C and then stained with indicated fluorochrome-conjugated antibodies (eBioscience) and a fixable viability dye (Life Technologies). Labeled cells were acquired BD LSRFortessa and data processed using FlowJo software (Treestar).

### Quantigene® gene expression analysis

Total RNA was isolated from snap frozen tumor tissue lysed using the MagMax™ 96 Total RNA isolation kit (Life Technologies) homogenized with steel beads on a TissueLyser (Qiagen) for 2 min at 25 Hz. Samples were processed washed, and incubated with DNase, on the MagMax™ Express 96 Processor. 500 ng of RNA was incubated in duplicate with QuantiGene® magnetic capture beads, probesets, and blocking reagent (Affymetrix) and analyzed on the FlexMap 3D® (ThermoFisher, Waltham, MA). Level of RNA detection was determined by mean fluorescence intensity (MFI) and converted into adjusted net MFI using an in-house quality control analysis script. “Net MFI”: sample was calculated as MFI – background MFI of blank well; “Adjusted Net MFI”, calculated: if MFI > lower limit of detection (LLOD, background MFI + 3 standard deviations), then “Adjusted Net MFI” = “Net MFI”, if MFI < LLOD, then “Adjusted Net MFI” = LLOD – background. Adjusted Net MFI was used to calculate relative gene expression normalizing each gene to the geometric mean of the MFI of selected housekeeping genes (HKG) (adjusted net MFI/geometric mean HKG MFI) multiplied by a scaling factor of 100. Data visualizations were done using TIBCO Spotfire® software (Spotfire, Somerville, MA).

## Results

### Galunisertib blocks TGFβ1 mediated suppression of naïve T cell proliferation and blocks Treg mediated suppression of naïve T cells

**Fig. 1:**
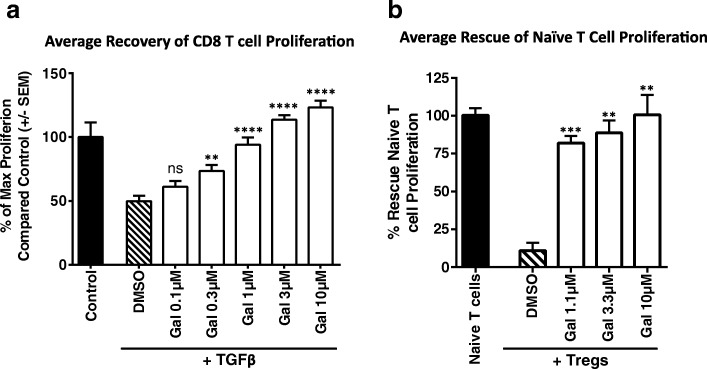
Galunisertib blocks TGFβ1 mediated suppression of naïve T cell proliferation and blocks Treg mediated suppression of naïve T cells. **a** Human CD8^+^ cells purified from healthy donor whole blood where labeled with CFSE and stimulated with anti-CD3/CD28 beads in the presence of TGFβ1 and galunisertib. CD8^+^ T cell proliferation was measured by assessing CFSE dilation by flow cytometry after 5 days of culture. Percent recovery of CD8^+^ T cell proliferation at different concentrations of galunisertib (0-10 μM) as indicated and representative histograms are shown. DMSO was used as vehicle control. Data shown are combined data of 4 healthy donors. **b** Human CD4^+^CD25^+^ cells purified from healthy donor whole blood where labeled with CFSE and stimulated with anti-CD3/CD28 beads in the presence of galunisertib. Autologous CD4^+^CD25^−^ Treg were added at a 1:1 ratio of Treg cells to naïve T cells. Naïve CD4^+^CD25^−^ T cell proliferation was measured by assessing CFSE dilation by flow cytometry after 5–7 days of culture. Percent recovery of CD4^+^CD25^−^ T cell proliferation at different concentrations of galunisertib (0-10 μM) as indicated and representative histograms are shown. Data shown are combined data of 4 healthy donors; representative of 3 independent experiments. One-way ANOVA with Dunnett’s test was used to compare the galunisertib treatments to the DMSO treatment. ****: *p* ≤ 0.0001; ***: *p* ≤ 0.001; **: *p* ≤ 0.01; *: *p* ≤ 0.05; ns: *p* ≥ 0.05

TGFβ signaling plays an important role in suppressing an immune reaction and inducing tolerance. In particular, TGFβ signaling inhibits innate and adaptive immune functions and induces suppressive immune cells. To test if galunisertib could rescue TGFβ suppressed immune cell subsets, naïve T cell suppression assays were established, and suppression mediated by TGFβ1 or by T regulatory cells (Tregs) was tested in in vitro culture systems. For these experiments, naïve human CD8^+^ T cells were stimulated with anti-CD3/anti-CD28 beads in the presence or absence of TGFβ1. As shown in Fig. 1a, while TGFβ1 potently suppressed the proliferation of CD8^+^ T cells, addition of galunisertib resulted in a dose-dependent rescue of proliferation in the TGFβ1 treated cultures, with enhanced proliferation observed at the higher doses of galunisertib. To evaluate the ability of galunisertib to modulate Treg suppressive activity, CD4^+^CD25^+^ Treg cells were co-cultured with naïve T cells (CD4^+^CD25^−^) in the presence of anti-CD3/anti-28/anti-CD2 stimulation. While CD4^+^CD25^+^ Tregs potently suppressed naïve T cell proliferation, addition of galunisertib fully reversed the suppression of proliferation, demonstrating a role for galunisertib in reversing Treg mediated immune suppression (Fig. 1b).

### Galunisertib monotherapy induces regression of immunogenic 4T1-LP tumors

**Fig. 2:**
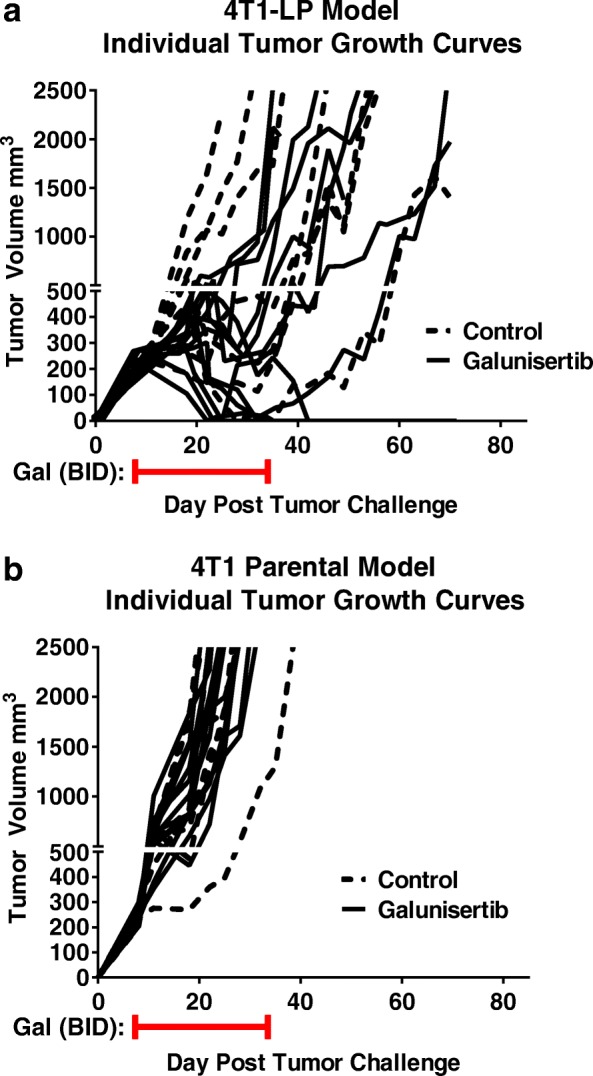
Galunisertib monotherapy induces regression of immunogenic 4T1-LP variant breast tumors. Mean and individual tumor growth curves for Balb/c mice injected orthotopically in the mammary fat pad with 4T1-LP (**a**) or parental 4T1 (**b**) tumor cells and treated with galunisertib (75 mg/kg BID) when tumors reached ~300mm^3^ (6–8 days after implantation). The number of mice/group rejecting tumors (complete responders, CRs) was: control (0/10 mice) and galunisertib (4/12) for 4T1-LP, and control (0/10 mice) and galunisertib (0/12) for 4T1 parental as indicated. Data shown are representative of two independent experiments with 10–12 mice/group

To explore the impact of galunisertib monotherapy on preventing growth of established tumors, we utilized the poorly immunogenic murine triple negative breast tumor model, 4T1, and a variant engineered to express luciferase, (4T1-LP). For these experiments, immune competent Balb/c mice were injected orthotopically in the mammary fat pad with 4T1-LP or 4T1 tumors. When tumors were well established (~300mm^3^, ~ 8–11 days after implantation), animals were treated with galunisertib at 75 mg/kg BID. Animals were treated for 28 days then followed for tumor growth. In the 4T1-LP model, the majority of mice (10/12) responded to galunisertib therapy, including 4/12 complete responses (Fig. 2a); in contrast, none of the poorly-immunogenic 4T1 bearing mice responded to galunisertib therapy (Fig. 2b), suggesting that the presence of a foreign antigen (i.e. LP), potentially enhanced the ability of galunisertib to induce the rejection of the 4T1-LP derivative. In a previous study, a survival benefit advantage with galunisertib was observed in the poorly-immunogenic 4T1 tumor model (Yingling et al., [25]). This may reflect the early start of treatment in that study (day 4 after tumor implantation compared to 8–11 days in the study presented here) or it may be a result of an anti-metastatic activity rather than an effect only on primary tumor growth.

A few mice in the vehicle control group in the 4T1-LP model, but not in the parental 4T1 model, showed an initial tumor response before eventually progressing (Fig. 2a), suggesting that spontaneous responses to immunogenic tumor cell lines can occur in some mice. This may reflect the different T cell repertoire between individual mice as we did not use TCR transgenic mice, or suggesting that these mice developed an immune response to a dominant CTL epitope of LP, which may lead to reduced tumor invasiveness and spontaneous regression.

[28]. Although, a few mice of the control group showed initial spontaneous activity, all untreated tumors eventually progressed without treatment. The spontaneous activity we observe in a few of the 4T1-LP control mice (Fig. 2a) is likely reflective of an immune response to the implanted tumors, and this immune response may in fact be the mechanism by which galunisertib is so much more active as a monotherapy in 4T1-LP compared to 4T1 parental. We speculate that the immunogenic nature of 4T1-LP is likely what impacts galunisertib monotherapy activity in this model, while in less immunogenic tumor models, combination with anti-PD-L1 is needed (described below).

**Fig. 3:**
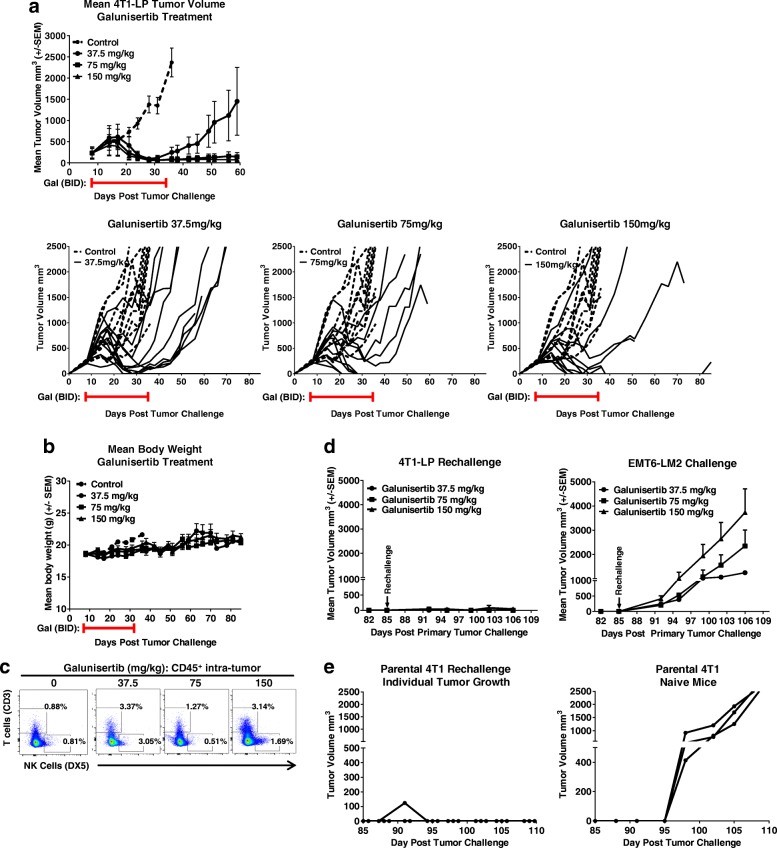
Galunisertib monotherapy displays dose dependent activity against established orthotopic 4T1-LP tumors with immunologic memory and antigen spreading. **a** Mean and individual tumor growth curves for Balb/c mice injected orthotopically in the mammary fat pad with 4T1-LP tumor cells and treated with galunisertib (37.5, 75 or 150 mg/kg BID) when tumors reached ~ 300 mm3 (8 days after implantation). The number of CRs were: 1/10, 3/10 and 5/10 for mice treated with Galunisertib at 37.5 mg/kg, 75 mg/kg and 150 mg/kg, respectively. **b** Percent body weight change on average of mice treated with galunisertib at 37.5 mg/kg, 75 mg/kg and 150 mg/kg. **c** Frequencies and representative plots of CD3^+^ and NK T cells of total live CD45^+^ cells in in single cell suspensions prepared from tumors harvested after 8 days of galunisertib treatment. Tumors from 5 mice/group treated with 75 mg/kg galunisertib or vehicle control were analyzed. Student’s t-tests were used to evaluate statistical significance (ns: *p* ≥ 0.05). **d** Mice which had regressed 4T1-LP tumors after treatment with galunisertib (37.5, 75 or 150 mg/kg BID for 28 days; as shown in **a**) were rechallenged orthotopically with 4T1-LP in one mammary fat pad and received a primary challenge of a different triple negative breast cancer tumor, EMT6-LM2, in an alternate fat pad, at day 85 post the original 4T1-LP tumor challenge. Average tumor growth curves of secondary 4T1-LP and EMT-6-LM2 challenge are shown for each group. **e** Mice which had regressed 4T1-LP tumors after treatment with galunisertib (75 mg/kg BID for 28 days) were rechallenged orthotopically with the poorly immunogenic 4T1 parental tumor cells at day 85 post the original 4T1-LP tumor challenge. Average tumor growth curves of secondary 4T1-LP challenge are shown. Individual tumor growth curves of naïve mice injected orthotopically with 4T1 parental tumors are shown as a control. Data shown are representative of two independent experiments with 10–12 mice/group

To further evaluate and interrogate the impact of galunisertib on anti-tumor activity, mice bearing 4T1-LP tumors in the mammary fat pad were treated for 28 days (starting at day 8 post tumor challenge) with three different doses of galunisertib (37.5, 75 and 150 mg/kg BID). Anti-tumor activity was observed at all 3 doses of galunisertib, with a dose dependent increase in activity as assessed by both mean tumor volume and CR (Fig. 3a-d). Anti-tumor activity was observed following an initial growth pattern similar to controls, indicating a delayed response to treatment and possible immune mediated mechanism. Following cessation of therapy on day 36 (28 days of treatment), responding mice progressed to complete responders in a dose-dependent manner, with 1/10 CR in mice treated with 37.5 mg/kg BID, 3/10 CRs in mice treated with 75 mg/kg BID, and 5/10 CRs observed in mice treated with 150 mg/kg BID (Fig. 3a). CR mice remained tumor free for an additional 49 days in the absence of further treatment. These data indicate that galunisertib induces a potent, dose-dependent durable anti-tumor response. Metastases to lungs were not observed in this tumor model.

Treatments with galunisertib were well-tolerated with no body weight loss observed with any of the doses tested (Fig. 3b).

Importantly, PK/PD profiling studies of galunisertib suggest that administration of 75 mg/kg BID in preclinical models or 150 mg/kg BID in patients can achieve significant target modulation in vivo over a 24-h period [1, 29]. Thus, we show anti-tumor activity with galunisertib at clinically relevant doses.

To begin to understand how galunisertib treatment modulated immune cells within the tumor, tumors from mice galunisertib treated or vehicle control mice were harvested 8 days after therapy initiation and the changes in T cell infiltration were analyzed by flow cytometry. Relative to control animals, a modest increase in both CD3 T cells, mainly CD8 T cells, and NK cells was observed in tumors of mice treated with the clinically relevant dose of 75 mg/kg galunisertib (Fig. 3c), indicating a role of galunisertib on T-cell expansion or T-cell trafficking to the tumor site. These differences did not reach statistical significance. No significant changes were observed in the myeloid compartment in tumors of galunisertib treated mice compared to control treated mice in this model. However, only the number of myeloid cells was analyzed and not the function; thus, whether galunisertib induces reprogramming toward an antitumor phenotype was not explored. This may also reflect the time point of tumor collection. To this end, a prior study with anti-mouse TGFβRII showed modulation of MDSCs by blocking the TGFβ signaling pathway [13].

### Galunisertib monotherapy induces immunologic memory and demonstrates antigen spreading

4T1-LP tumor bearing mice that completely responded to galunisertib therapy remained tumor free for up to 85 days (49 days after treatment completed) (Fig. 3a), indicating a durable response. To test the ability of galunisertib to induce immunologic memory to 4T1-LP tumors, mice which had completely regressed 4T1-LP were re-challenged orthotopically with 4T1-LP on the opposite flank of the original tumor injection site and additionally received a primary challenge of a different triple negative breast cancer tumor, EMT6-LM2, on the flank of the original tumor injection site. In all mice tested, complete responders rejected the re-challenge with the 4T1-LP tumors (Fig. 3d, left panel), but did not reject EMT6-LM2 tumors (Fig. 3d, middle panel), demonstrating immunologic memory to the 4T1-LP tumor cells, but not the heterologous tumor. To evaluate the potential for epitope spreading as a result of galunisertib anti-tumor activity, mice which had completely regressed 4T1-LP after being treated with 75 mg/kg of galunisertib were re-challenged in a separate experiment with the poorly immunogenic parental 4T1 tumors, which lack the immunogenic LP transgene and is not responsive to de-novo galunisertib monotherapy (Fig. 2b); in all mice tested, 4T1-LP complete repressors also rejected the parental 4T1 challenge (Fig. 3e, right panel), demonstrating the potential for galunisertib anti-tumor activity to mediate antigen spreading.

### Galunisertib anti-tumor activity in the 4T1-LP model is CD8 T cell dependent

**Fig. 4:**
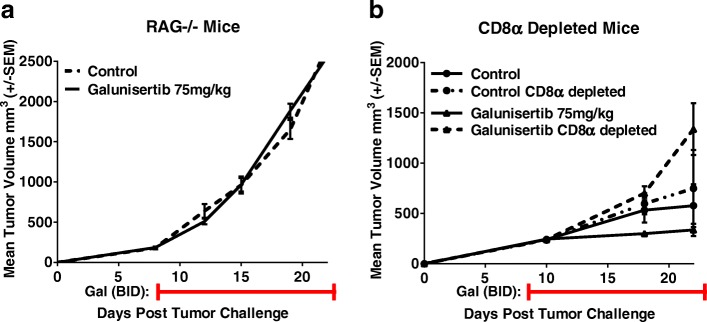
Galunisertib anti-tumor activity in the 4T1-LP model is CD8 T cell dependent. **a** Mean tumor growth curves for Rag^−/−^ mice injected orthotopically in the mammary fat pad with 4T1-LP tumor cells and treated with galunisertib (75 mg/kg BID × 28 days) when tumors reached ~ 300 mm3 (8 days after implantation). **b** Mean tumor growth curves for Balb/c mice injected orthotopically in the mammary fat pad with 4 T1-LP tumor cells and treated with galunisertib (75 mg/kg BID × 28 days) plus depleting antibody for CD8α (200 μg/mouse). Treatment was initiated when tumors reached ~ 300 mm3 (8 days after implantation). CD8α monoclonal antibody was injected on day 1, 2 and 3 after tumor challenge, followed by injection weekly throughout the experiment. Data represent two independent experiments with 8 mice/group

The delayed response to galunisertib and the modest increase in tumor-infiltrating lymphocytes (TILs) in treated mice suggested that the adaptive immune response may be involved in the mechanism of tumor rejection following galunisertib therapy. To evaluate the role of the adaptive immune response in galunisertib anti-tumor activity, studies using the orthotopic 4T1-LP model were carried out in the RAG^−/−^ mice or in Balb/c mice depleted of CD8^+^ T cells by treatment with an immune depleting anti-CD8α antibody. In both RAG^−/−^ and CD8^+^ T cell depleted mice bearing 4T1-LP tumors, galunisertib therapy was unable to induce regression of tumors indicating a requirement for an adaptive immune system, and in particular CD8^+^ T cells, in this model (Fig. 4).

### Combined blockade of TGFβR1 and PD-L1 enhnaces regression of tumors

**Fig. 5:**
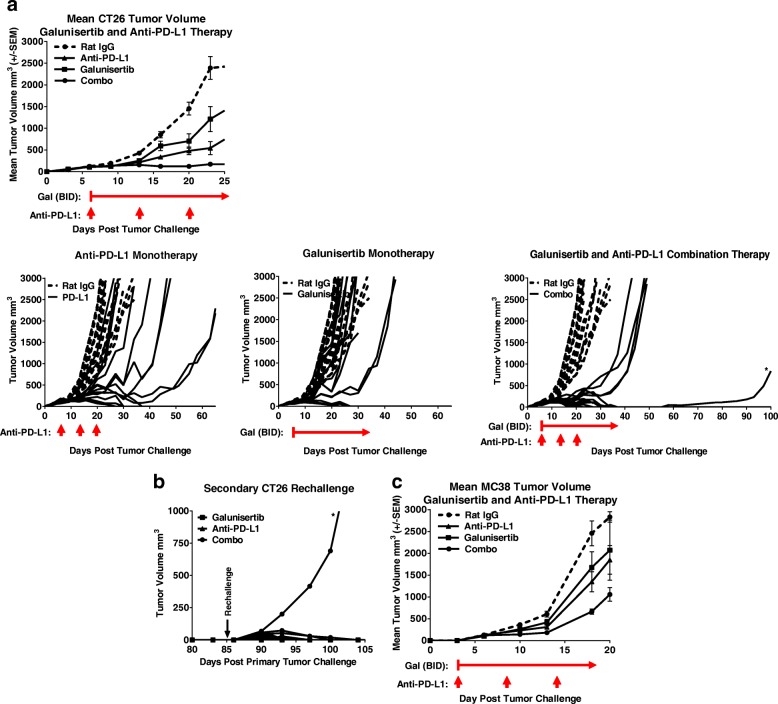
Combined blockade of TGFβR1 with Galunisertib and PD-L1 induce robust regression of murine colon tumors. Mean and individual tumor growth curves for Balb/c mice injected with CT26 tumor cells and treated with galunisertib (75 mg/kg BID for 28 days) and/or anti-PD-L1 antibody (500 μg/dose, q7dx3) when tumors reached ~100mm^3^ (6 days after implantation). The percentages of CRs were: control (0/15 mice), galunisertib monotherapy (3/14), anti-PD-L1 monotherapy (5/15) and combination therapy (9/14). **b** Mice which had regressed CT26 tumors after treatment with galunisertib and/or anti-PD-L1 were rechallenged with CT26 tumor cells on the contralateral flank at day 85 post the original tumor challenge. Individual tumor growth curves of secondary CT26 challenge are shown for each treatment group. **c** Mean and individual tumor growth curves for C57BL/6 mice injected with MC38 tumor cells and treated with galunisertib (75 mg/kg BID for 21 days) and/or anti-PD-L1 antibody (500 μg/dose, q7dx3). Treatment was initiated 3 days after tumor cell implantation. Data shown are representative of two independent experiments with 10–12 mice/group

Because the in vitro and in vivo galunisertib monotherapy data strongly suggested that galunisertib was able to modulate anti-tumor T cell immunity, we investigated whether galunisertib could synergize with PD-L1 checkpoint blockade and result in improved tumor regressions. Anaphylactic reactions have been reported with PD-L1 and PD1 monoclonal antibodies in the orthotropic 4T1 tumor model [30] (Mall et al., [31]), thus, for these studies we utilized the CT26 mouse model well known to be responsive to various degrees to PD-1 axis immunotherapy [32]. Balb/c mice were injected with CT26 tumor cells and treated with galunisertib, anti-PD-L1 or a combination of both for 21 days. Treatment was initiated on Day 6 when tumors were ~100mm^3^ and continued for 28 days. The mean tumor growth under each condition is presented in Fig. 5 (left panel), and shows that while both galunsertib and anti-PD-L1 therapy were active in this model, the combination of galunisertib and anti-PD-L1 resulted in enhanced anti-tumor activity. As shown in the individual animal plots, anti-PD-L1 or galunisertib monotherapy were modestly active in this setting with 5/15 and 3/14 CR respectively while the combination of anti-PD-L1 and galunisertib therapy resulted in marked enhancement of response (9/14 CRs) and all animals responding to treatment (Fig. 5). There was a significant antitumor benefit with the combination group versus each monotherapy (*p* < .001). To test if each treatment could result in immunologic memory, mice with CRs were re-challenged with CT26 tumors 85 days after primary tumor challenge (51 days after initial treatment cessation). All complete responders in monotherapy and combination treatment groups rejected the re-challenge with the CT26 tumors (Fig. 5b, left panel); however, one animal from the combination cohort, defined as a long-term partial responder (indicated by * in Fig. 5a right panel) was unable to reject secondary tumor challenge. The ability of galunisertib to enhance the activity of anti-PD-L1 immunotherapy was confirmed in the MC38 tumor model, which is historically less responsive to checkpoint immunotherapy and considered to be more myeloid biology driven [32]. In this model, where treatment began on day 3 after tumor challenge, similar monotherapy and combination therapy activity was observed albeit with more moderate activity overall (Fig. 5c).

### Combination of galunisertib and anti-PD-L1 checkpoint blockade induces an intra-tumor immune related gene expression profile that is accelerated and enhanced compared to anti-PD-L1 monotherapy

**Fig. 6:**
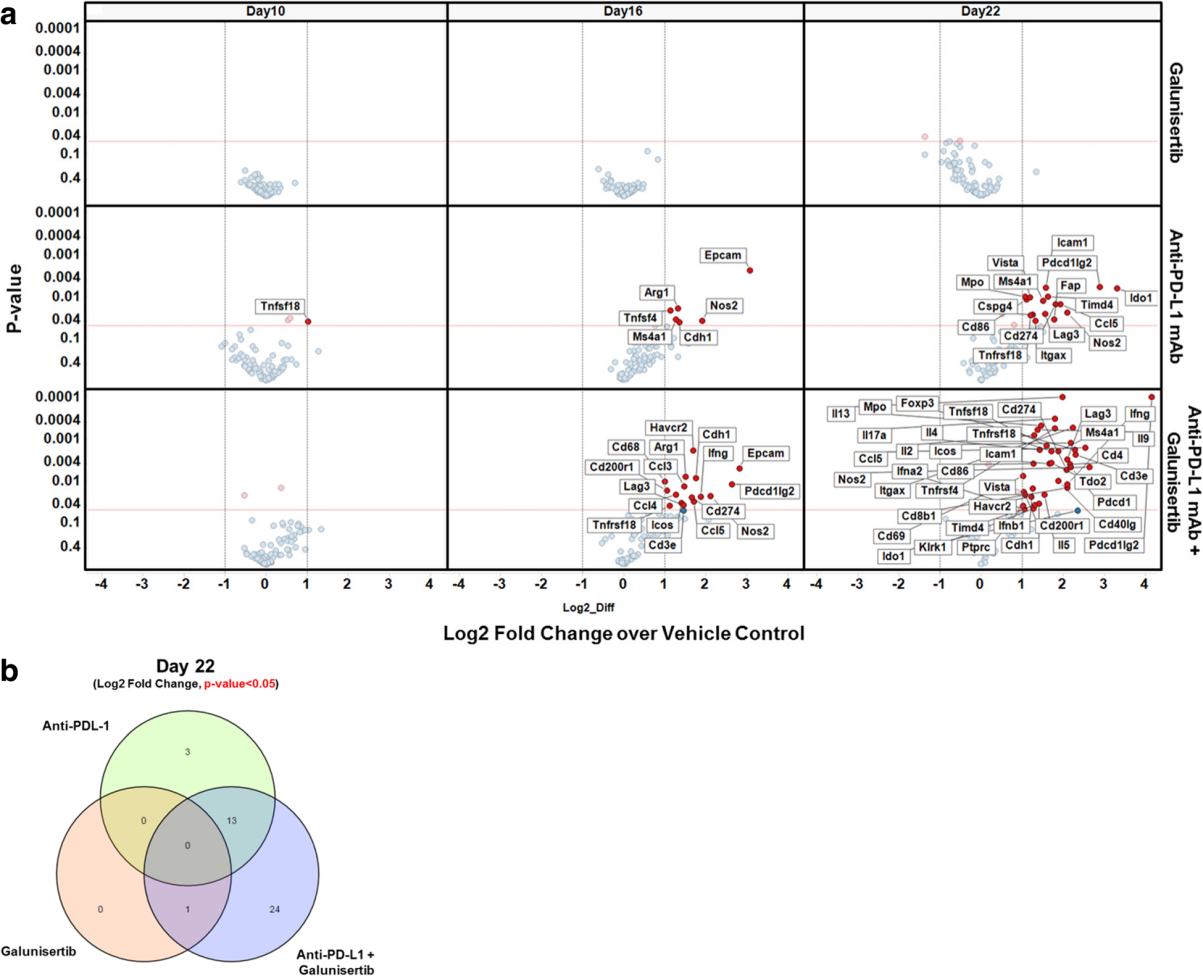
Combination of galunisertib and anti-PD-L1 checkpoint blockade induces an intra-tumor immune related gene expression profile that is accelerated and enhanced compared to anti-PD-L1 monotherapy. Log2 Fold Change in gene expression of galunisertib (75 mg/kg BID for 28 days) and/or anti-PD-L1 (500 μg/dose, q7dx3) treated CT26 tumors against vehicle control. Differential expression and *p*-value was evaluated by two-way ANOVA testing. Labelled and highlighted genes are log2FC > 1, p-value< 0.05, are shown in Volcano Plots (**a**) and Venn Diagrams (**b**). Tumors were harvested and analyzed at different time points after tumor challenge: day 10, 16 and 22 (treatments were initiated at 6 days after implantation)

To further elucidate the mechanism of action of the combination activity of galunisertib and anti-PD-L1, gene expression studies were carried out on tumors from CT26 tumor bearing mice treated with control, anti-PD-L1, galunisertib or a combination of anti-PD-L1 plus galunisertib. For these studies, tumors from treated mice were collected day 10, 16, and 22 after tumor challenge (i.e. 4, 10, and 16 days after initiation of therapy) and subjected to high-content molecular profiling using a custom designed Quantigene^TM^ gene panel to detect T cell activation and intra-tumoral inflammation (Table 1). Galunisertib monotherapy (75 mg/kg) did not appreciably alter the set of immune genes analyzed relative to control tumors at any time point evaluated (Fig. 6, top panel). Anti-PD-L1 monotherapy resulted in an enhanced T cell infiltration and activation profile, exemplified by the increase in multiple immune activation and inflammation transcripts such as Ccl5, Itgax, Icam1, Foxp3, Lag3 by day 22 (Fig. 6a). On the other hand, the combination treatment demonstrated an early signature of enhanced T cell activation and inflammation exemplified by the upregulation of transcripts for Ifnγ, Lag3, Ccl3, Ccl4, Ccl5, and Tnfrsf18) beginning on Day 16 (after only 10 days of therapy, where only a minor change in activation was detected with anti-PD-L1 monotherapy), and continuing at day 22 with an enhanced gene expression related to T cell infiltration (Ptprc, Cd8b1, Cd3e, and Cd4) and T cell activation and inflammation (Il2, Il4, Il17a, Lag3, Ifnγ, Ifnα, Ifnβ1, Foxp3, Cd274, and Pdcdlg2) (Fig. 6a). The gene profile for the combination cohort was similar but more robust compared to PD-L1 monotherapy detected at the later time point (day 22) (Fig. 6a and b). Interestingly, the treatments also resulted in increased expression of some immunosuppressive genes, such as Ido1, Mpo, Nos2 and Tdo2, which may reflect a counter-regulatory mechanism induced by the tumor and/or myeloid cells in response to enhanced IFNγ production by anti-PD-L1 or combination treated tumor-infiltrating T cells. In support of this, preclinical work using murine tumor models have shown that dual targeting of IDO and checkpoints results in enhanced anti-tumor immunity [33–35]. Overall, combination therapy resulted in an accelerated and more robust increase in genes indicative of T cell activation compared to either monotherapy suggesting that inhibiting immune suppression with galunisertib may accelerate the biological activity of anti-PD-L1. Examination of immune cell subset frequencies in tumors by flow did not detect major changes during therapy, and T cell frequencies were similar for monotherapy PD-L1 and combination therapy (data not shown), suggesting that the effects of combination were modulated at the effector function level. Finally, we observed that some genes such as FAP were upregulated at day 22 upon anti-PD-L1 treatment but not with combination therapy, suggesting that galunisertib may be acting by remodeling tumor stroma (Fig. 6a), as previously described in the literature with other inhibitors of the TGFβ pathway [13, 36].

**Table 1:** Custom designed Quantigene™ gene panel to detect T cell activation and intra-tumoral inflammation

Cell type-specific markers	Coinhibitory & Costimulatory	Cytokines & Chemokines	Immunosuppressive Enzymes	Markers of T cell activation	Angiogenesis; Endothelial activation	EMT markers	TGFb pathway	HKGs
*Cd14*	*4632428N05Rik (Vista)*	*Ccl2 (MCP-1)*	*Arg1*	*Cd69*	*Cdh5*	*Cdh1*	*Smad4*	*Gus*
*Cd3e*	*Cd200r1*	*Ccl3 (MIP-1a)*	*Ido1*	*Gzmb*	*Hif1a*	*Epcam*	*Tgfb1*	*Hprt*
*Cd4*	*Cd274 (PD-L1)*	*Ccl4 (MIP-1b)*	*Mpo*	*Ifng*	*Vcam1*	*Fap*	*Tgfb2*	*Ppib*
*CD68*	*Cd40lg*	*Ccl5 (RANTES)*	*Nos2*		*Vegfa*	*Snai1*	*Tgfb3*	*Rps18*
*Cd8b1*	*Cd86*	*Csf2 (GM-CSF)*	*Tdo2*		*Vegfc*	*Twist1*	*Tgfbr1*	
*Foxp3*	*Havcr2 (TIM3)*	*Cxcl1*			*Icam1 (CD54)*	*Vim*	*Tgfbr2*	
*Itgam (CD11B)*	*Icos*	*Ifna2*			*Sele*	*Cspg4*		
*Itgax (CD11C)*	*Lag3*	*Ifnb1*						
*Klrk1*	*Pdcd1 (PD-1)*	*Il10 (CSIF)*						
*Ms4a1*	*Pdcd1lg2 (PD-L2)*	*Il13*						
*Ptprc (CD45)*	*Pvr*	*Il17a*						
	*Pvrl2*	*Il2*						
	*Tigit*	*Il4*						
	*Timd4 (TIM4)*	*Il5*						
	*Tnfrsf18 (GITR)*	*Il6*						
	*Tnfrsf4 (OX40)*	*Il9*						
	*Tnfrsf9 (4-1BB)*	*Tnf*						
	*Tnfsf18 (GITRL)*							
	*Tnfsf4*							
	*Tnfsf9 (CD137L, 4-1BBL*)

## Discussion

TGFβ plays diverse and important roles in tumorigenesis, including tumor proliferation, invasion and metastasis, inflammation, angiogenesis, and escape of immune surveillance. Therefore, targeting the TGFβ signaling pathway has been an attractive objective for cancer therapy, and several drugs have been identified and are under clinical development [37–39]. Galunisertib is a small molecule inhibitor of the kinase domain of TGFβRI. Binding of TGFβ ligands to TGFβRII is the first step to initiate activation of the TGFβ signaling pathway. Once bound to TGFβRII, this ligand/receptor complex binds to TGFβRI to form a heterotrimeric complex. Formation of this complex results in phosphorylation of the serine/threonine kinase domains of the receptors, followed by activation of the canonical SMAD2/3 signaling pathways as well as non-canonical (i.e. MAPK) pathways [39]. These pathways modulate transcription of numerous target genes, resulting in a variety of effects. By blocking the kinase domain of TGFβRI, galunisertib may effectively inhibit signaling via the TGFβ pathway.

Preclinical and clinical research on galunisertib, including the treatment of over 800 patients, has demonstrated that SMIs of TGFβ can safely be developed for clinical testing, provided there is an adequate understanding of the pharmacokinetic/pharmacodynamics (PK/PD) relationship, as most of the toxicities in animal models that were of concern prior to the start of clinical development of galunisertib have not been observed in humans [1]. Furthermore, preclinical and clinical efforts suggest that the biology of the TGFβ inhibition is largely dependent on the microenvironment, perhaps more than originally anticipated. For example, TGFβ1 is a potent inducer of angiogenesis [40], by directly inducing VEGF expression [41], or recruiting other cells, such as monocytes, which in turn secrete proangiogenic molecules [42]. TGFβ can also manipulate the tumor microenvironment to favor the evasion of cancer cells from immune surveillance via tampering with the antitumor functions of T cells, NK cells, B cells, and other cells [43–45]. This activity of TGFβ may be mediated through its direct effect on these cells, as well as via its ability to induce Foxp3^+^ Tregs [46]. Both cancer-intrinsic and immune-mediated effect of TGFβ in breast cancers have been described [47–49]. Thus, a focus on direct tumor cell cytotoxicity may be misleading and provide inconclusive observations that will not be helpful to advance clinical development of future TGFβ inhibitors. Early studies using immune-compromised animals may therefore also have limited the screening for TGFβ inhibitors. It now appears that an active immune response is essential to assess the effect of TGFβ signaling inhibition in animal models; thus, immune-competent animal models may be more predictive to evaluate TGFβ inhibitors. Consequently, more novel preclinical testing assays are required than those traditionally used in oncology research.

Here we describe the impact of galunisertib to modulate the immune system and its ability to enhance anti-tumor activity in immune-competent murine tumor models. We show that galunisertib monotherapy induces dose-dependent regression of well-established immunogenic 4T1-LP breast tumors. The responses were durable with immunological memory as demonstrated by rechallenge experiments with 4T1-LP tumors as well as a second triple negative breast tumor cell line. Of note, mice that rejected the immunogenic 4T1-LP tumors were also able to reject 4T1 parental cells upon rechallenge, suggesting the development of a secondary immune response via antigen spreading as a consequence of effective tumor targeting. The anti-tumor activity of galunisertib in the 4T1-LP tumor model was CD8^+^ T cell dependent and associated with a modest increase in T-cell infiltration in tumors. The increase was modest and did not reach statistical significance though, which might reflect the time point chosen for tumor collection. It is well established in the immuno-oncology field that the spatial distribution and location of immune cells is highly important. In fact, the recent publications combining TGFβ inhibition and PD-L1 blockade show that the main mechanism of action of TGFβ inhibition is to increase T-cell infiltration into tumor [26, 27]. In addition, using an anti-TGFβRII blocking antibody we have previously shown that blocking TGFβ signaling in the EMT6 tumor model induces immune infiltration [13].

We did not  investigate metastasis to lungs in either the 4T1 or 4T1-LP tumor models used here. However, we have previously shown that the anti-mouse TGFβRII antibody significantly inhibits the growth of established 4T1-parental primary tumors and diminishes the spontaneous pulmonary metastasis [13]. In addition, it was shown that galunisertib in combination with anti-CTLA4 therapy suppresses both primary melanoma tumor growth as well as metastases in a physiological relevant trangenic melanoma model (Hanks et al. [50]). Furthermore, we described that galunisertib inhibits TGFβ mediated migration of U87MG glioblastoma cells in vitro in a dose-dependent manner [51]. Notably, in this model system, galunisertib reduced baseline migration of U87MG cells in the absence of exogenous TGFβ1, presumably by inhibiting autocrine signaling through TGFβRI. Together this suggests that galunisertib has the capacity to suppress the development of metastasis and that TGFβ pathway blockade of the parental 4T1 model is sufficient to inhibit metastasis to lung.

Importantly, we have previously shown that the TGFβ pathway is abrogated upon treatment with galunisertib both in vitro and in vivo [51]. We demonstrated that galunisertib inhibited TGFβ-induced pSMAD in various tumor cell lines, including 4 T1-LP, in vitro in a dose-dependent manner [51]. Furthermore, we reported a galunisertib time and dose-dependent inhibition of endogenous TGFβ-dependent signal transduction in vivo in EMT6-LM2 murine syngenic tumor models [51]. These data suggest that the effects of galunisertib are on-target. Potential off-target effects of galunisertib are further diminished, as treatment with an anti-mouse TGFβRII antibody similarly inhibits the growth of established mouse 4T1 and EMT6 primary [13].

Immunotherapeutic strategies such as immune checkpoint blockade have shown significant promise for treatment of cancers resistant to conventional modalities, leading to Food and Drug Administration (FDA) approval in advanced melanoma, renal cell carcinoma and non-small cell lung cancer (NSCLC) [52]. Despite clinical results, even with combined checkpoint blockade [53], therapeutic success has so far been limited to a subset of patients, calling for identification of markers predicting response, identification of resistance mechanisms and development of combinatorial therapeutic approaches. To this end, TGFβ pathway inhibition represents an attractive strategy with its multitude of effects on cancer progression and on the immune system to enhance the development of anti-tumor T-cell immunity. Indeed, a recent study by Powles et al., reports that lack of response to atzeolizumab (anti-PDL1) in bladder cancer patients was associated with an immune-excluded phenotype that corresponded with active TGFβ in peritumoral stroma and a signature of TGFβ signaling [26]. Using mouse models that recapitulate the immune-excluded phenotype they further show that co-administration of blocking antibodies to TGFβ and PDL1 reduced TGFβ signaling, facilitated T-cell penetration of tumors, and provoked vigorous anti-tumor immunity leading to tumor regressions. In a second recent study published by Batlle and colleagues, combinatorial activity of galunisertib with anti-PDL1 in murine colon cancer models was recently described [27]. Combination therapy induced pronounced immune responses which eradicated most metastases, prolonged recurrence-free survival, and was associated with disruption of a T-cell exclusion phenotype. These results suggest that clinical co-administration of TGFβ and PDL1 blocking agents may provide a subset of patients more favorable outcomes; however, preclinical validation was performed with either a research-grade reagent [26] or a significantly excessive amount of galunisertib (800 mg/kg BID compared to the clinically relevant dose of 75 mg/kg described in [1, 29]. In agreement, we demonstrate that combination of galunisertib with PD-L1 checkpoint blockade results in a robust regression of CT26 tumors when compared to single agents. The observed antitumor benefit was associated with enhanced expression of genes indicative of immune activation and this gene expression profile was accelerated compared to anti-PD-L1 monotherapy. Galunisertib alone resulted in no alteration of any of the tested genes. Considering the critical role of TGFβ in cancer immunity, we speculate that this may be a result of the gene panel tested, the dose chosen or the day of collection. Similar combination therapy activity was observed in the PD-L1 insensitive tumor model, MC38, albeit with more moderate activity overall, suggesting at least additive activity with potential for synergy when targeting the TGFβ and PD-1 pathways. The anti-tumor activity of galunisertib was tested in a broad range of murine tumor models with similar results, further suggesting that TGFβ inhibition is immune mediated and thus not restricted to specific tumor indications.

Finally, we show that galunisertib reverses both TGFβ and Treg mediated suppression of T cell proliferation in human cell cultures in vitro, which further highlight the important role of galunisertib to overcome immune suppression and promote anti-tumor immunity.

Taken together, the results presented here demonstrate the impact of blocking TGFβ signaling and provide a strong incentive to clinically explore the potential of galunisertib treatment to enhance the development of anti-tumor T cell immunity, which may be enhanced by combinations with immune checkpoint inhibitors. Our results expand on other reports demonstrating that systemic treatment with monoclonal antibodies targeting the TGFβ ligands or the TGFβRII inhibit metastatic invasion of breast cancer cells in murine tumor models [2, 13], and previous work reporting that blocking TGFβ signaling with SMIs suppresses metastasis in murine pancreatic cancer models [54], and enhances radiation response and prolongs survival in xenograft models of glioblastoma [55].

Galunisertib continues to advance in clinical trials having completed Phase I [56] and is currently under investigation in several Phase I and Phase II trials. Thus far, galunisertib has been very well-tolerated as a first-in-class, oral cancer therapy, and remains a promising compound in clinical development (http://clinicaltrials.gov/ct2/results?term=LY2157299). Our data presented here support continued clinical development of galunisertib to target tumors dependent on TGFβ-driven biology for growth, metastasis, and immune evasion. Whether TGFβ inhibition applies to all tumors is not clear at this time. For clinical development, patient selection tools, defining who will most likely benefit from TGFβ inhibition, remain a challenging question. Among others, the activity of TGFβ inhibition appears to be dependent on immune function; thus, it will be important to investigate new biomarkers that are related to immune responses which may help with patient selection in future studies.

## Conclusion

In many advanced cancers, TGFβ ligands are overexpressed and the outcome of signaling is diverted toward disease progression. A concerted effort has therefore been to develop drugs that block TGFβ signaling for therapeutic benefit. Galunisertib is a pharmacological small molecule inhibitor of the TGFβ pathway that acts by inhibiting signaling through TGFβ receptor I. As a monotherapy, galunisertib has shown some antitumor activity in a variety of tumors, including durable and long-term responses in patients with glioma. Here, we demonstrate the ability of galunisertib to modulate anti-tumor T cell immunity, alone and in combination with PD-L1 checkpoint blockade, in preclinical models. Our data provide a strong rationale to clinically explore the potential of galunisertib to enhance anti-tumor immune response, particularly, in combinations with PD-L1/PD-1 checkpoint inhibitors. Galunisertib is currently under clinical development in combination with checkpoint inhibitors (including nivolumab and durvalumab) in patients with NSCLC, HCC, or pancreatic cancer.
